# Synergistic EMI Shielding Efficiency of Biodegradable PLA/PBS-Based Composites Reinforced with Carbon Fibers and ZnO Nanoparticles

**DOI:** 10.1021/acsomega.6c02664

**Published:** 2026-06-03

**Authors:** Necla Altin, Saeid Darvishi, Gulsen Kurt Demir, Alp Oral Salman, Ayse Aytac

**Affiliations:** † Department of Chemical Engineering, Engineering Faculty, 52980 Kocaeli University, Kocaeli 41001, Türkiye; ‡ Department of Electronics and Communication Engineering, Engineering Faculty, 52980 Kocaeli University, Kocaeli 41001, Türkiye; § Polymer Science and Technology Department, Kocaeli University, Kocaeli 41001, Türkiye

## Abstract

The escalating electromagnetic pollution from modern electronics, combined with the global plastic waste crisis, demands the development of high-performance EMI shielding materials based on sustainable and biodegradable polymers. Biodegradable polyesters such as polylactic acid (PLA) and polybutylene succinate (PBS) offer a compelling solution owing to their renewable origin, low carbon footprint, and susceptibility to enzymatic degradation by proteases, lipases, and esterases under composting conditions, which enable responsible end-of-life disposal and support circular economy principles. In this context, this study focuses on the development of biodegradable PLA/PBS-based composites with exceptional electromagnetic interference (EMI) shielding effectiveness through the synergistic combination of carbon fibers (CF) and ZnO nanoparticles. A fixed CF content of 20 wt % was employed to establish a conductive backbone, while ZnO nanoparticles were incorporated at 1, 3, 5, and 7 wt % to enhance dielectric loss and absorption-based shielding. EMI shielding measurements in the X-band revealed a dramatic improvement in total shielding effectiveness (SET), increasing from 1.7 dB for the PLA/PBS matrix to 30.7 dB with CF reinforcement and reaching 70, 59, 73, and 85 dB for composites containing 1, 3, 5, and 7 wt % ZnO, respectively. The composite containing 1 wt % ZnO exhibited the most efficient EMI shielding response relative to filler content, achieving a high SET of ∼70 dB with minimal nanoparticle loading. This superior performance was attributed to the uniform dispersion of ZnO nanoparticles, which maximized interfacial polarization, dielectric loss, and multiple internal scattering within the carbon fiber-supported network. Structural, rheological, thermal, and mechanical analyses consistently showed that increasing ZnO content beyond 1 wt % led to nanoparticle agglomeration, network disruption, reduced melt elasticity, and mechanical embrittlement, despite further increases in absolute SET at higher loadings. EMI shielding in all composites was dominated by absorption (SEA > 80%), making them particularly suitable for practical EMI mitigation applications. These findings demonstrate that optimized ZnO dispersion at low loading is a key factor in achieving efficient, absorption-dominant EMI shielding in biodegradable polymer composites, offering a sustainable and environmentally responsible alternative to conventional metal-based and petroleum-derived polymer shielding materials.

## Graphical abstract

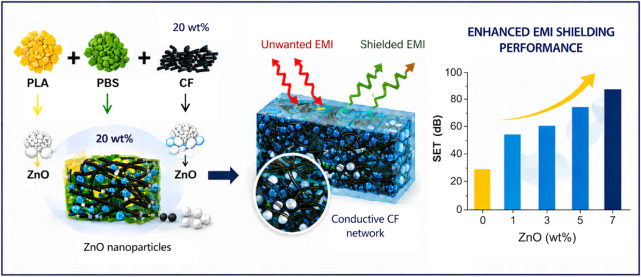

## 1 Introduction

The rapid technological advancements and the proliferation of electronic devices, wireless communication systems, and high-frequency telecommunications (5*G*/6G) have revolutionized human connectivity and efficiency.^1^ However, this progress has inadvertently escalated electromagnetic interference (EMI), a form of pollution that disrupts electronic equipment, compromises data integrity, and poses potential health risks through prolonged exposure to electromagnetic radiation.^2–5^ EMI arises from the unintended emission, transmission, and reception of electromagnetic energy, particularly in the X-band (8–12 GHz) and Ku-band (12–18 GHz), which can degrade the performance of sensitive electronics in sectors such as aerospace, automotive, telecommunications, healthcare, and consumer electronics.^6,7^ Traditional EMI shielding materials, predominantly metals like aluminum, copper, nickel, and their alloys, offer high shielding effectiveness (SE > 60 dB) due to their excellent electrical conductivity and ability to reflect electromagnetic waves via impedance mismatch.^8,9^ However, these materials suffer from several critical drawbacks, including high density (2.7–8.9 g/cm^3^), susceptibility to corrosion in humid environments, poor flexibility, electrical grounding requirements, and high processing costs, which limit their applicability in lightweight, portable, and sustainable applications.^10^

As global awareness of environmental sustainability intensifies—coupled with the plastic waste crisis (400+ million tons annually)—there is a pressing need for eco-friendly EMI shielding alternatives that maintain or exceed the performance of conventional materials while reducing carbon footprints, enabling end-of-life recyclability, and achieving biodegradability under composting conditions.^11,12^ Polymer-based composites have emerged as promising candidates for next-generation EMI shielding due to their inherent advantages: low density (<1.5 g/cm^3^), corrosion resistance, ease of processing via melt extrusion or 3D printing, and tunable electromagnetic/mechanical properties through filler engineering.^13,14^ Among these, biodegradable polymers stand out for addressing the dual challenges of EMI pollution and plastic waste accumulation.^12^ Biodegradable polyesters such as PLA and PBS are particularly attractive for sustainable EMI shielding applications, as they can be degraded through both hydrolytic and enzymatic pathways. PLA is susceptible to enzymatic attack by proteinase K, lipases, cutinases, and esterases, which cleave the ester backbone into lactic acid monomers readily assimilated by microorganisms.^15^ Similarly, PBS undergoes enzymatic hydrolysis by lipases under both industrial composting and soil-burial conditions.^16^ This intrinsic biodegradability ensures that EMI shielding components based on PLA/PBS do not persist as microplastic pollution after end-of-life disposal, aligning with circular economy and green electronics principles.

Polylactic acid (PLA) is a leading biodegradable polyester known for its biocompatibility, high stiffness (Young’s modulus ∼3.5 GPa), transparency, and moderate thermal stability (*T*_g_ ≈ 60 °C, *T*_m_ ≈ 165 °C).^17,18^ PLA degrades via hydrolysis and enzymatic action in 6–24 months under industrial composting, making it ideal for sustainable applications.^19^ However, pure PLA exhibits critical limitations: brittleness (elongation at break <6%), low impact resistance (<10 kJ/m^2^), slow crystallization, and poor heat deflection temperature (<60 °C), hindering its use in EMI shielding applications.^20,21^

To overcome these shortcomings, blending PLA with polybutylene succinate (PBS)—another biodegradable aliphatic polyester—has proven highly effective.^22,23^ PBS complements PLA by providing superior toughness (elongation > 300%), flexibility, and thermal processability (*T*_m_ ≈ 115 °C) while maintaining overall biodegradability.^24^ PLA/PBS blends (typically 70/30 or 80/20 wt %) exhibit synergistic mechanical performance: increased elongation at break (20–150%), impact strength, and balanced crystallinity due to partial miscibility, phase separation morphology, and interfacial interactions that promote stress transfer.^22,25^ These blends retain biodegradability and are processed via conventional melt mixing, ensuring uniform dispersion without solvents or complex chemical modifications.^26^

To impart EMI shielding capabilities to PLA/PBS matrices, conductive fillers are essential for forming percolating networks that enable electron transport, dielectric loss, and multiple internal reflections for wave attenuation.^27^ Carbon fiber (CF), with its exceptional properties such as high electrical conductivity, tensile strength, and Young’s modulus, is an ideal primary reinforcement for polymer composites.^28^ CF-reinforced polymers demonstrate enhanced shielding through dual mechanisms: reflection (via high conductivity) and absorption (via fibrous interfaces and multiple scattering).^29^ In biodegradable systems, ∼20 wt % CF loadings boost electrical conductivity, tensile modulus, and EMI SE.^30^ However, high CF loadings often cause agglomeration, reduced processability, increased viscosity, and compromised biodegradability.^31,32^

To achieve superior shielding efficiency while minimizing the total filler content and preserving matrix ductility, hybrid filler systems incorporating semiconducting nanoparticles have gained significant traction.^33,34^ Zinc oxide (ZnO) nanoparticles enhance EMI shielding performance through several complementary mechanisms. They promote dielectric loss by facilitating interfacial polarization and dipole relaxation, strengthen overall conductivity by forming synergistic bridges within the carbon fiber (CF) network, and improve filler dispersion through electrostatic stabilization.^35,36^ In hybrid systems, low ZnO loadings achieve superior EMI shielding due to the morphological advantage compared to the binary composite.^37^

Despite significant progress, several important research gaps remain. Studies on fully biodegradable quaternary systems such as PLA/PBS/CF/ZnO are still limited, and the synergistic interactions between ZnO and carbon fibers at optimized ratios have not yet been fully explored. Comprehensive data on absorption-dominant shielding performance are also lacking, and achieving a shielding effectiveness above 70 dB in entirely biodegradable composites remains rare.^12,38^

This study addresses these gaps by systematically investigating PLA/PBS blends reinforced with 20 wt % CF and varying ZnO nanoparticle contents (0, 1, 3, 5, 7 wt %), prepared via melt-mixing. Through rigorous multitechnique characterization, we demonstrate exceptional EMI SE ≈ 80 dB—surpassing most reported biodegradable composites—while maintaining mechanical integrity, thermal stability, and full biodegradability. To the best of our knowledge, this is the first systematic study on a fully biodegradable quaternary PLA/PBS/CF/ZnO composite system for EMI shielding applications. The unique aspects and contributions of this work can be summarized as follows: (i) it is the first report on a fully biodegradable quaternary PLA/PBS/CF/ZnO system achieving exceptional EMI SE of 70–85 dB, far exceeding most biodegradable composites (typically 15–42 dB); (ii) it reveals that ZnO dispersion quality at low loading (1 wt %) is more critical than filler quantity for efficient shielding; (iii) the absorption-dominant mechanism (SEA > 80%) minimizes secondary electromagnetic pollution, unlike typical reflection-dominant CF composites; and (iv) the entirely biodegradable matrix addresses the growing e-waste crisis. These findings establish PLA/PBS/CF/ZnO as a sustainable, high-performance platform for next-generation EMI shielding in electronics, aerospace, and beyond.

## 2 Materials and Methods

### 2.1 Materials

PLA and PBS polymers were used as matrix materials. 4043D poly­(lactic) acid that has a molecular weight of 120,000 g/mol was supplied from Nature Works (USA). PLA has a density of 1.24 g/cm^3^ and a melting temperature of 155 °C. PBS (Mw: 104,000 g/mol) was obtained from PTT MCC Biochem Co., Ltd. under the trade name BioPBS FZ71PM, with a density of 1.26 g/cm^3^ and a melting temperature of 115 °C. PA-coated chopped carbon fiber has a bulk density of 575 g/L and was obtained from DowAksa (Yalova, Türkiye). Zinc Oxide (ZnO) (Nanografi, Türkiye) with 99.5% purity, a density of 5.5 g/cm^3^, and a particle size of 30–50 nm was purchased.

### 2.2 Processing (Extruder)

The PLA/PBS (80/20, wt %/wt %) blend and PLA/PBS/CF/ZnO nanocomposites containing 20 wt % CF and 1, 3, 5, and 7 wt % ZnO nanoparticles were prepared via melt compounding using a corotating twin-screw micro compounder (MC 15 HT, Xplore Instruments). Before the melt-mixing methods, polymer granules were vacuum-dried at 80 °C for 12 h to remove residual moisture and prevent hydrolytic degradation during extrusion.

During compounding, PLA, PBS granules, and ZnO nanoparticles were fed simultaneously into the compounder, followed by melt blending for 2.15 min residence time, at a barrel temperature of 180 °C and a screw speed of 60 rpm. After achieving a homogeneous melt, CF was fed into the compounder and blended for 45 s to prevent excessive fiber breakage while ensuring uniform dispersion.

Following melt compounding, the extrudates were molded using a micro injection molding unit (MI 12, Xplore Instruments) to produce specimens for tensile test following the ISO 527-2 (5A) standard and rectangular sheets (4 cm × 8 cm × 2 mm) for EMI shielding characterization. The injection molding operation was carried out under an injection pressure of 10 bar, whereas the melt and mold temperatures were set at 180 and 25 °C.

### 2.3 Characterization

Structural properties were determined with Fourier transform infrared spectroscopy (FTIR) by using PerkinElmer Spectrum 100. Measurements were performed in the range of 650–4000 cm^–1^ wavelength.

The mechanical properties of the samples at room temperature were determined by a tensile test. The tests were performed using dog-bone specimens at a tensile speed of 5 mm/min according to the ISO 527-5A standard. Tensile tests were repeated five times for each sample, and the results are given as average values.

Rheological measurements in the melt state were performed using an Anton Paar MCR 102 rheometer equipped with a parallel geometry with a diameter of 25 mm. For small-amplitude oscillatory shear (SAOS) measurements at 180 °C, the first amplitude sweep test was performed from 0.001% to 10% strain at 10 1/s frequency to find the linear viscoelastic region (LVR). The frequency sweep measurements were then performed between 0.1 and 600 1/s at LVR.

The thermal stability of the PLA/PBS/ZnO–CF composite was evaluated using thermogravimetric analysis (TGA, Mettler Toledo TGA 1). In a nitrogen atmosphere, samples in the range of 5–10 mg were analyzed at temperatures ranging from 25–600 °C and a heating rate of 10 °C/min. The measurements determined the samples’ 5% (Td5), 10% (Td10), and 50% (Td50) decomposition temperatures, maximum decomposition temperature (*T*_max_), and residual weights.

Thermal transition behaviors were investigated using the Mettler Toledo Star DSC1 differential scanning calorimeter. Measurements were conducted at a heating rate of 10 °C/min under a nitrogen atmosphere. In the applied thermal program, the samples were heated from −50 to 200 °C at a rate of 10 °C/min, followed by a 2 min isothermal hold. Subsequently, a second heating cycle from −50 to 200 °C was applied at the same heating rate.

The microstructural properties of the composites were analyzed by using a scanning electron microscope (SEM, QUANTA 400 F). Before observation, the samples were coated with a thin layer of gold (Au) to enhance the conductivity. To obtain cross-sectional images, the samples were mechanically fractured to create a suitable cross-sectional surface, thereby enabling the examination of the internal morphology.

The EMI shielding performance of samples in the 8.2–12.4 GHz frequency range was determined using a two-port vector network analyzer (Rohde & Schwarz ZVB20 VNA) equipped with a WR-90 waveguide system (Figure 1). Prior to the measurements, a two-port full calibration was performed on the coaxial cable ends, and the TOSM (through, open, short, match) calibration method was applied. In the experiments, injection-molded samples with dimensions of 4 cm × 8 cm × 2 mm (thickness) were used. To calculate the EMI shielding effectiveness (SE), the scattering parameters (S-parameters) S11 (input reflection at port 1) and S21 (transmission from port 1 to port 2) were obtained from the VNA device. A schematic representation of the S-parameters is shown in Figure 1a, and the EMI SE measurement setup is given in Figure 1b.

**Figure 1:**
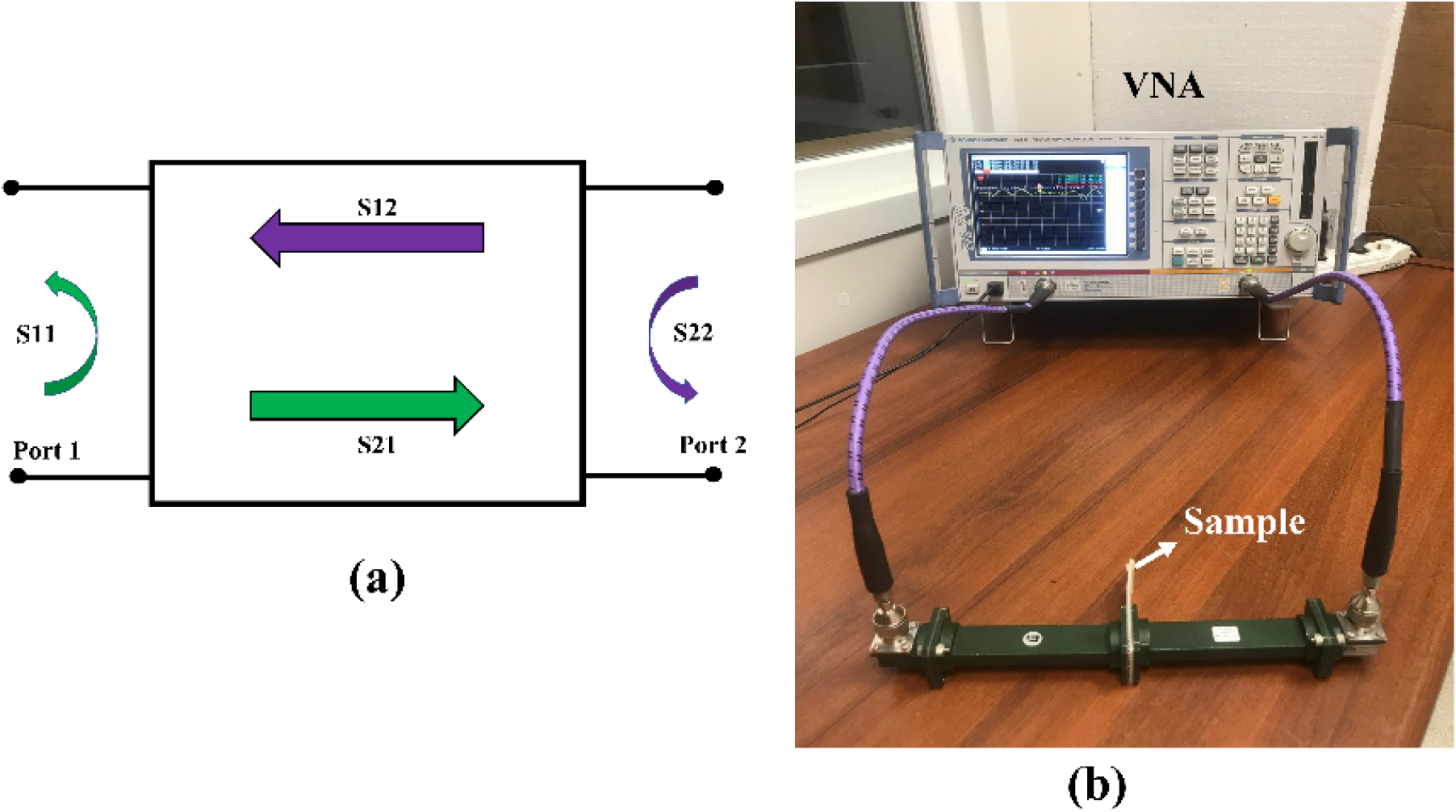
(a) Schematic representation of scattering parameters and (b) EMI SE measurement setup.

To evaluate the EMI shielding performance of composites, scattering parameters (S11 and S12) were measured; using these data, transmitted power (*T*), absorbed power (*A*), and reflected power (*R*) were calculated. The total EMI shielding effectiveness (SE_T_) includes reflection (SE_R_), absorption (SE_A_), and multiple internal reflection (SE_M_). However, SE_M_ is generally neglected when the total EMI shielding effectiveness exceeds 10 dB. The SE_A_, SE_R_, and SE_T_ values were obtained using the following equations. Here, *R*, *T*, and *A* represent the reflection coefficient, transmission coefficient, and absorption coefficient, respectively^39–41^

1 $$R={|S_{11}|}^{2}$$

2 $$T={|S_{21}|}^{2}$$

3 1=A+R+T

4 $${\mathrm{SE}}_{R}=-\mathrm{10 log}\left(1-R\right)$$

5 $${\mathrm{SE}}_{A}=\mathrm{-10 log}\left(\frac{T}{1-R}\right)$$

6 $${\mathrm{SE}}_{T}{=\mathrm{SE}}_{R}{+\mathrm{SE}}_{A}{+\mathrm{SE}}_{M}$$

7 $$A_{\mathrm{eff}}\;\left(\%\right)=A/\left(1-R\right)\times 100$$

## 3 Results and Discussion

### 3.1 SEM Results

The fracture surface morphologies of the PLA/PBS blend and PLA/PBS/CF composites containing 3, 5, and 7 wt % ZnO nanoparticles are shown in Figure 2. SEM analyses were realized to evaluate the fracture morphology of composites, fiber–matrix interfacial interactions, and the contribution of the fiber-reinforced structure to the overall fracture mechanism. The neat PLA/PBS blend exhibits a relatively smooth and homogeneous fracture surface with limited plastic deformation. This morphology is characteristic of PLA-dominant biodegradable blends, in which restricted chain mobility results in a predominantly brittle fracture behavior. Owing to its limited capacity for plastic deformation and the formation of flat fracture surfaces, the intrinsic brittle fracture character of the PLA/PBS blend was considered as a reference state in the present analysis. The nanometer-scale size of ZnO nanoparticles, which fall below the resolution limit of conventional SEM, together with their low contrast within the polymer matrix, prevents their direct identification. This limitation has been explicitly reported in the literature as a common issue in nanoparticle-reinforced polymer systems.^42–44^ Therefore, morphological interpretations regarding the structural role of ZnO were deliberately not based on SEM data.

**Figure 2:**
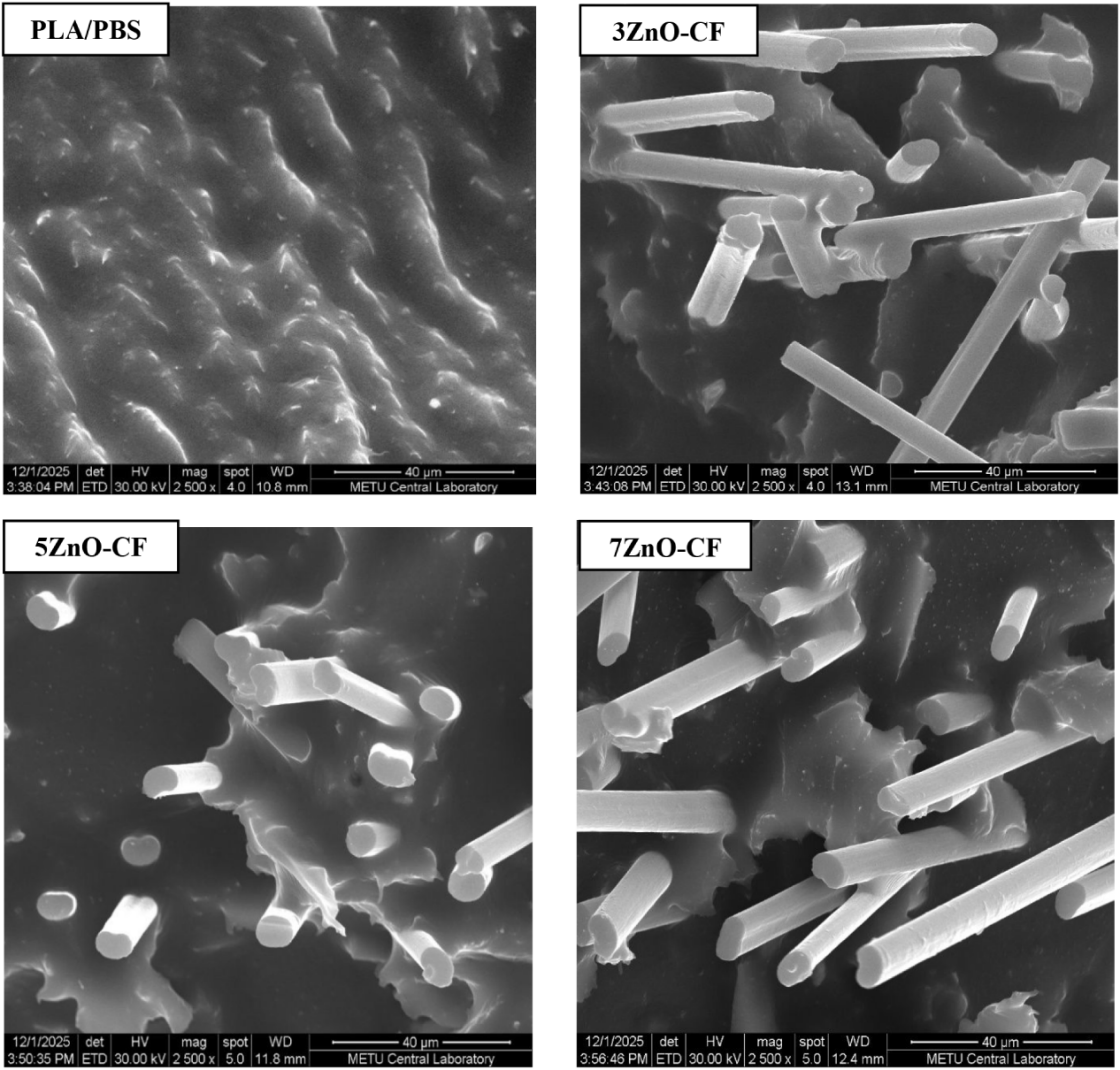
SEM images of PLA/PBS, 3ZnO–CF, 5ZnO–CF, and 7ZnO–CF (magnification: × 2500).

SEM micrographs indicate that the fracture surfaces of all composites containing ZnO and carbon fibers are dominated by a fibrous morphology, with the fracture mechanism progressing primarily along the fiber–matrix interface. The absence of any noticeable differences in carbon fiber geometry, distribution characteristics, or fiber-specific morphological features among the composites containing 3, 5, and 7 wt % ZnO demonstrates that the incorporation of ZnO does not directly modify the carbon fiber architecture. All elongated and fibrous structures observed in the micrographs correspond to carbon fibers, and no micron-scale secondary phase or particle clusters attributable to ZnO nanoparticles were detected. Similar findings have been reported in previous studies on short carbon fiber-reinforced PLA and PLA/PBS-based biodegradable composites, where fracture behavior was shown to be predominantly governed by fiber–matrix interactions.^30,45^

Within this context, the effect of ZnO nanoparticles on the composite structure was evaluated not through SEM morphology, but through rheological measurements, which are directly sensitive to nanoparticle presence and interactions. It is widely accepted in the literature that rheological analyses can reveal nanoparticle-induced network formation, filler–filler interactions, and percolation behavior with much higher sensitivity than SEM.^44,46^ In particular, the pronounced increase in storage modulus (*G*′) and complex viscosity (η*) observed (see Figure 6) at low ZnO content (1 wt %) indicates that the carbon fiber-supported network structure becomes more effectively interconnected in the presence of ZnO. This behavior reflects the strengthening of filler–filler and filler–matrix interactions that cannot be directly visualized by SEM but can be detected with high sensitivity through rheological measurements.^44,46^

As the ZnO content increases, the observed deterioration in the rheological parameters suggests a disruption of effective network continuity and a gradual weakening of the load-bearing structure. This trend is commonly associated with the tendency of ZnO nanoparticles to agglomerate at higher loadings, leading to a reduction in the effective interaction surface area. Similar rheological behaviors have been reported for ZnO-containing PLA and PLA/PBS systems.^42,43,46^ These results indicate that the influence of ZnO nanoparticles on the internal structural organization of the composites can be more reliably elucidated through rheological measurements rather than through direct morphological observations.

### 3.2 FTIR Results of ZnO–CF Reinforced PLA/PBS Composites

Figure 3 presents the FTIR spectra of the neat PLA/PBS (80/20) blend, the PLA/PBS/CF composite, and PLA/PBS/CF nanocomposites containing 1, 3, 5, and 7 wt % ZnO nanoparticles, recorded in the range of 3700–600 cm^–1^. All samples exhibit the characteristic absorption bands of the PLA/PBS matrix, including the ester C=O stretching at ∼1748 cm^–1^, C–O–C stretching vibrations at ∼1180, 1082, and 1042 cm^–1^, −CH_3_ deformation bands at ∼1452 and 1386 cm^–1^, and −CH stretching near 2960 cm^–1^. The positions of these bands remain unchanged after the incorporation of carbon fibers and ZnO nanoparticles. No new absorption bands or peak shifts are observed upon filler addition, indicating that the chemical structure of the PLA/PBS matrix is preserved and that interactions among the matrix, carbon fibers, and ZnO nanoparticles are predominantly physical. With increasing ZnO content, a gradual increase in intensity and broadening of the O–H stretching region (3600–3200 cm^–1^) and a weak band near 1630–1600 cm^–1^, attributed to surface hydroxyl groups and adsorbed moisture on ZnO, are observed. All CF-containing composites display a shoulder at ∼1360 cm^–1^ that is absent in the neat PLA/PBS blend. This feature is assigned to O–H bending vibrations of phenolic hydroxyl groups on the oxidized carbon fiber surface^47^ and remains nearly constant across all ZnO loadings, confirming the uniform incorporation of carbon fibers.

**Figure 3:**
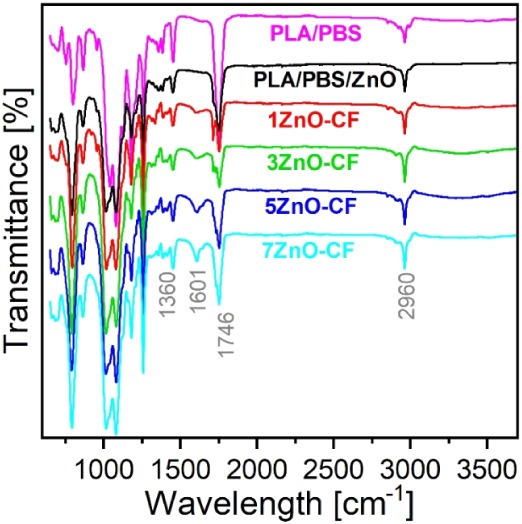
FTIR spectra of the neat PLA/PBS (80/20) blend and PLA/PBS/CF/ZnO nanocomposites containing 1, 3, 5, and 7 wt % ZnO nanoparticles.

### 3.3 Thermal Analysis (DSC and TGA) of Composites

The thermal analysis data obtained from the DSC thermograms of the first and second heating cycles of the samples are presented in Figure 4 and Table 1, respectively. The *T*_g_ value of ∼55 °C determined in the PLA/PBS (80/20) composite indicates that the glass transition behavior is largely controlled by PLA due to its high ratio, and that the mixture’s *T*_g_ is consistent with the typical *T*_g_ of PLA.^48^ The *T*_g_, *T*_m1_, *T*_m2_, and Δ*H*_m1_, Δ*H*_m2_ values were determined in the first and second heating cycles of the composites, and dual melting peaks corresponding to PBS and PLA were detected. In the DSC analysis of PLA/PBS composites, the melting temperature of the PLA phase was observed to be approximately 155 °C, while that of the PBS phase was approximately 115 °C.^49,50^ The partial decrease in *T*_g_ and changes in melting enthalpy observed with increasing ZnO content indicate that the addition of ZnO influences the crystallization behavior of the polymer matrix. Cruz Faria et al. reported that ZnO particles slightly shifted the glass transition temperature in the PLA matrix.^51^ Similarly, Jamnongkan et al. reported that changes in *T*_g_ and *T*_m_ values in ZnO-containing PLA filaments were limited.^44^ In this study, the decrease observed in *T*_g_ with increasing ZnO content may be due to ZnO particles creating different free volume and chain mobility effects on polymer chains.

**Figure 4:**
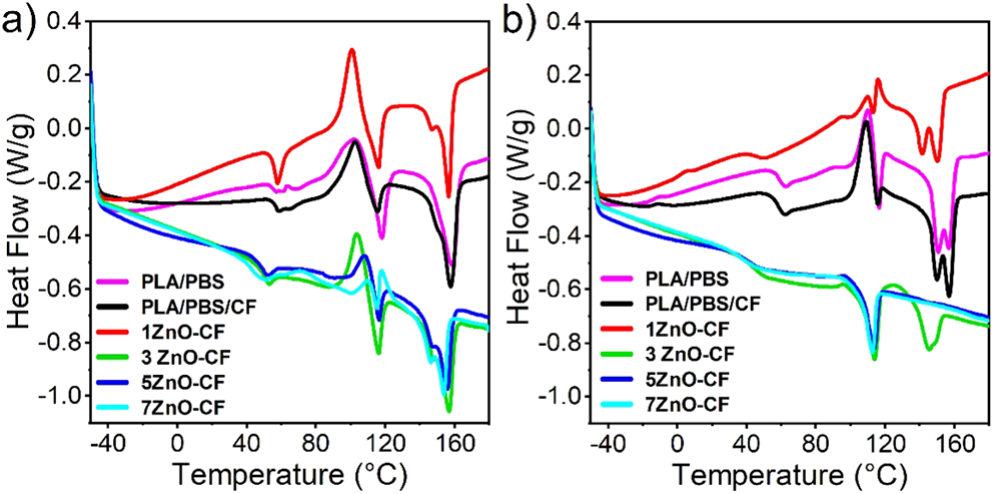
DSC thermograms of the composites (a) First Heating and (b) Second Heating.

**Table 1:** Thermal Transition Temperatures of the Samples

	**First Heating**					**Second Heating**			
Sample	*T*_g_ (°C)	*T*_m1_ (°C)	*T*_m2_ (°C)	Δ*H*_m1_ (J/g)	Δ*H*_m2_ (J/g)	*T*_m1_ (°C)	*T*_m2_ (°C)	Δ*H*_m1_ (J/g)	Δ*H*_m2_ (J/g)
**PLA/PBS**	55.28	118.04	158.49	16.61	20.34	116.24	156.68	4.53	20.49
**PLA/PBS-CF**	57.24	115.41	157.63	1.24	21.72	115.58	156.78	0.81	23.13
**1 ZnO–CF**	51.15	116.12	156.75	8.63	17.86	113.02	150.29	0.49	17.71
**3 ZnO–CF**	50.05	115.71	156.59	14.92	9.19	113.54	144.86	13.50	9.68
**5 ZnO–CF**	49.31	116.11	155.48	5.70	14.55	113.07	-	10.01	-
**7 ZnO–CF**	43.56	114.95	153.64	2.62	5.33	112.24	-	9.26	-

It is reported that carbon fiber reinforcement has no significant effect on *T*_g_, but it can increase the degree of crystallinity of the composite through a heterogeneous nucleation mechanism.^52,53^ Furthermore, it is generally accepted that the differences between the first and second heating curves are due to cold crystallization and enthalpic relaxation processes occurring during the first heating.^51^ The obtained DSC data, consistent with the findings of previous studies, indicate that ZnO addition can affect the thermal transitions and crystallization behavior of PLA/PBS composites, whereas carbon fibers primarily direct the formation of the crystalline structure.

EMI shielding materials may heat up when exposed to electromagnetic waves during use. Therefore, thermal stability is critical for EMI applications and the melting process of polymer composite materials. For this reason, the thermal stability of PLA/PBS/CF-ZnO samples was investigated using TGA. TGA and derivative thermogravimetry (DTG) curves are presented in Figure 5a–b, while the thermal decomposition data of the samples are presented in Table 2. Upon examination of the TGA curves, the PLA/PBS blend and CF/PLA/PBS composites decomposed in a single step, while the ZnO–CF-doped samples decomposed in two steps. Previous studies have also reported that a fixed 20% carbon fiber (CF) content increases the thermal stability of the polymer matrix and raises its residue.^45,54,55^ Our results show that the degradation onset temperature increases and the residual solid content rises in CF-modified PLA/PBS composites. This effect can be explained by the stabilization of the internal structure due to the high thermal stability of the fibers.^56^ Therefore, the fixed 20% CF has transformed the matrix into a structure that degrades relatively slowly even without ZnO, and when combined with the effect of ZnO, it has supported the observed stable high heat resistance. It is observed that the initial mass loss temperatures (*T*_deg5_ and *T*_deg10_) decrease significantly with the addition of ZnO and an increase in the additive ratio. Virág and Molnár reported that adding ZnO to the PLA/PBS matrix reduced the onset of thermal degradation.^46^ The initial decomposition temperature for pure PLA/PBS (80/20) is ∼319 °C, while adding 7% ZnO reduces it to ∼235 °C. This is consistent with the study reporting that the initial decomposition temperature of ∼310 °C found by Virág and Molnár for the PLA/PBS matrix decreased to 236 °C with the addition of 10% ZnO.^46^ This similar trend indicates that the effect of ZnO nanoparticles in reducing the thermal stability of the polymer matrix depends on both the ZnO ratio and the nature of the polymer–filler interactions. In summary, increasing ZnO content reduces the thermal stability of the PLA/PBS/CF composites, indicating that degradation begins at lower temperatures.

**Figure 5:**
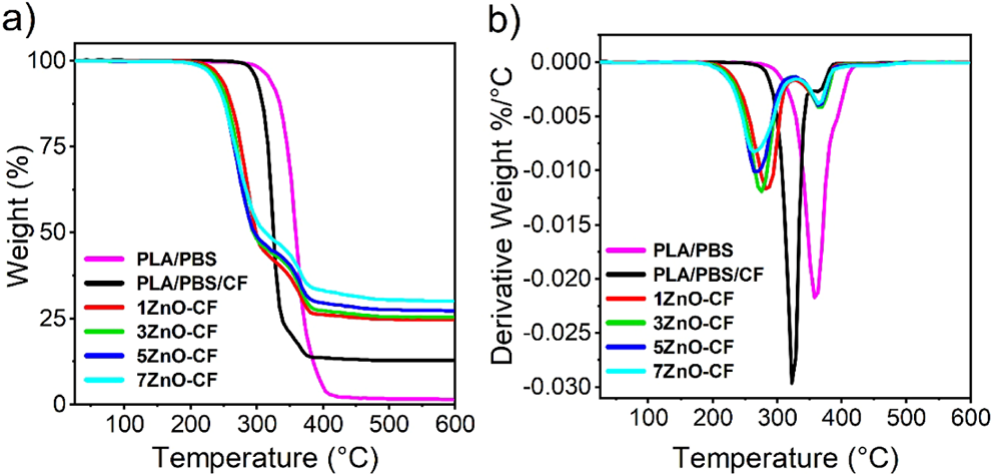
(a) TGA and (b) DTG results of samples.

**Table 2:** Thermal Degradation Data of the Samples

Sample	*T*_deg5_ (°C)	*T*_deg10_ (°C)	*T*_deg50_ (°C)	*T*_max1_ (°C)	*T*_max2_ (°C)	Residue Amount (%, 600 °C)
**PLA/PBS**	318.71	330.92	359.05	359.83	-	1.56
**PLA/PBS-CF**	299.06	306.52	325.52	324.37	364.70	12.88
**1 ZnO–CF**	245.19	257.56	298.56	283.49	367.33	24.78
**3 ZnO–CF**	239.30	251.87	294.73	274.55	366.38	25.41
**5 ZnO–CF**	234.99	247.66	296.77	267.29	365.57	27.32
**7 ZnO–CF**	234.93	247.56	313.55	263.29	364.70	30.07

It is noteworthy that thermal decomposition in ZnO-doped composites occurs in two stages, resulting in two distinct peaks observed in the TGA and DTG curves. When examining the DTG curves, a single peak (around 359 °C) is observed in the sample without ZnO, while two distinct peaks appear in the composites containing 1–7% ZnO. The first of these two peaks (∼263–274 °C) most likely corresponds to the first-stage decomposition of the PLA matrix, while the second (∼364–366 °C) corresponds to the PBS component or secondary decomposition. As the amount of ZnO increases, the first peak temperature decreases slightly, while the second peak remains relatively constant. These results indicate that ZnO-modified PLA/PBS/CF composites undergo a two-step degradation process and that ZnO facilitates the primary degradation. Besides, it is observed that the remaining residue percentage increases significantly as the ZnO and CF content increases. This is consistent with ZnO and CF remaining independent of molecular decomposition at high temperatures. The residual content in 20% glass fiber-reinforced PLA/PBS composites has increased from 4.56% to 15.82%.^55^ The resulting TGA data are consistent with other studies in the literature. It has been determined that ZnO promotes thermal decomposition, while CF enhances heat resistance.

### 3.4 Mechanical and Rheological Test Results

Rheological characterization was performed on the PLA/PBS/CF composite and PLA/PBS/CF composites containing 1, 3, 5, and 7 wt % ZnO nanoparticles via frequency sweep tests within the linear viscoelastic region. Figure 6a presents the evolution of the storage modulus (*G*′) and loss modulus (*G*″) as functions of angular frequency for PLA/PBS/CF composites containing different ZnO nanoparticle loadings. The unmodified PLA/PBS/CF composite exhibits a pronounced solid-like viscoelastic response, characterized by *G*′ values consistently exceeding *G*″ over the entire frequency range. Moreover, both moduli show weak frequency dependence at low frequencies, which is indicative of a well-developed, percolated carbon fiber network that effectively restricts polymer chain mobility and enables efficient stress transfer within the melt. The incorporation of a low ZnO nanoparticle content (1 wt %) leads to a remarkable enhancement in the viscoelastic module, with *G*′ and *G*″ increasing by approximately 2 orders of magnitude compared to the ZnO-free composite. This pronounced reinforcement effect can be attributed to the uniform dispersion of ZnO nanoparticles within the PLA/PBS matrix at low loading levels.^46^ Well-dispersed nanoparticles act as additional physical cross-linking points and promote interfacial interactions with the polymer chains and carbon fibers, thereby strengthening the filler–matrix network and enhancing the elastic and viscous responses of the composite. In contrast, further increases in ZnO content result in a progressive decline in both *G*′ and *G*″, accompanied by a stronger dependence of the moduli on angular frequency. At higher nanoparticle loadings, the viscoelastic moduli fall below those of the unmodified PLA/PBS/CF composite. This behavior suggests the onset of ZnO nanoparticle aggregation, which disrupts the continuity of the filler network and reduces the effective surface area available for polymer–filler interactions. The formation of aggregates diminishes stress transfer efficiency and weakens the physical network structure, leading to a reduction in melt elasticity. At the highest ZnO loading investigated (7 wt %), the aggregation of nanoparticles becomes dominant, resulting in the formation of large ZnO clusters.^46^ Under these conditions, the composite exhibits a transition toward viscous liquid-like behavior, as evidenced by *G*″ surpassing *G*′ across the measured frequency range and the strong frequency dependence of both moduli. In parallel with our observation in thermal analysis (reducing *T*_g_ value by increasing ZnO content), this rheological response reflects a breakdown of the percolated network and a substantial increase in polymer chain mobility, consistent with a system governed primarily by viscous flow rather than elastic deformation.

**Figure 6:**
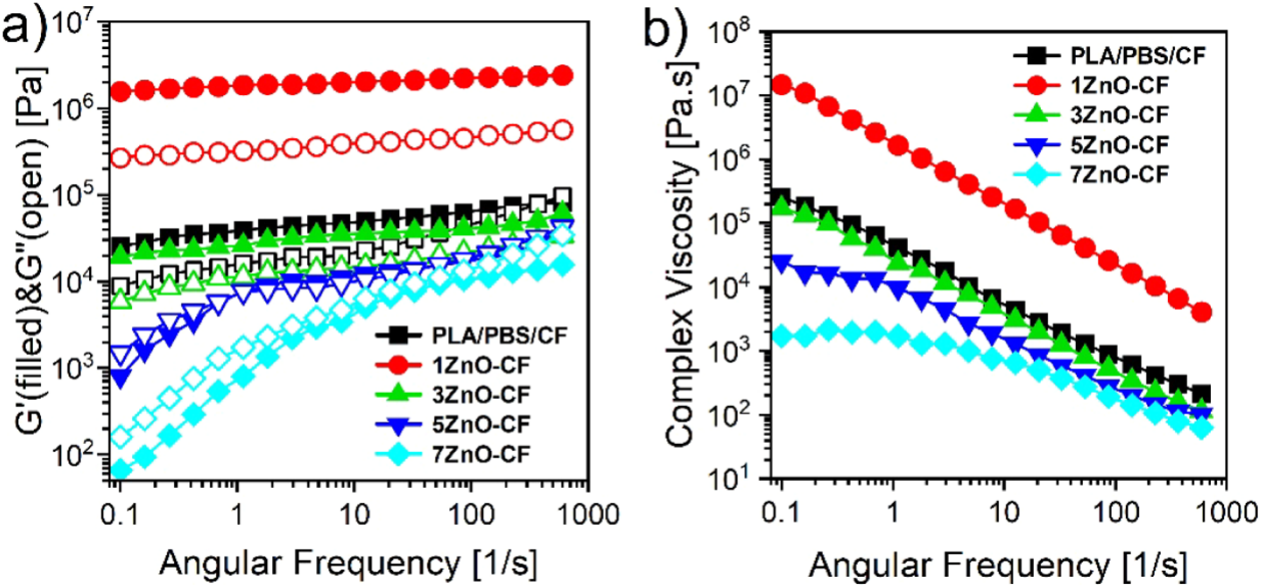
(a) Storage modulus (*G*′) and loss modulus (*G*″) as a function of angular frequency for PLA/PBS/CF composites with varying ZnO nanoparticle loadings (0, 1, 3, 5, and 7 wt %). (b) Complex viscosity (η*) versus angular frequency for the same composites, illustrating the influence of ZnO content on melt rheological behavior and shear-thinning characteristics.

Figure 6b illustrates the variation of complex viscosity (η*) as a function of angular frequency for the same set of PLA/PBS/CF composites containing different ZnO nanoparticle loadings. Among all formulations, the composite incorporating 1 wt % ZnO exhibited the highest complex viscosity across the entire frequency range, followed by the unmodified PLA/PBS/CF composite. This behavior is consistent with the dynamic moduli results (Figure 6a) and confirms the formation of a strong, interconnected filler–matrix network at low ZnO content. The enhanced viscosity at 1 wt % ZnO can be attributed to the homogeneous dispersion of nanoparticles, which increases hydrodynamic volume and intensifies polymer–filler and filler–filler interactions, thereby significantly restricting polymer chain mobility. As the ZnO nanoparticle loading increased to 3, 5, and 7 wt %, the complex viscosity decreased systematically. This reduction in η* reflects the progressive deterioration of the effective filler network due to nanoparticle agglomeration. The formation of ZnO aggregates lowers the interfacial contact area between the polymer matrix and the fillers, weakens stress transfer efficiency, and disrupts the percolated structure established by carbon fibers. Consequently, the melt experiences reduced resistance to flow, leading to lower viscosity values at higher ZnO contents. All composites exhibited pronounced shear-thinning behavior over the tested frequency range, indicating the breakdown of physical networks and alignment of polymer chains and fillers under increasing shear. This non-Newtonian response is characteristic of filled polymer melts with transient, shear-sensitive microstructures. Notably, the PLA/PBS/CF composite containing 7 wt % ZnO displayed a distinct deviation from this trend by exhibiting a near-Newtonian viscosity plateau at low frequencies (<1 Hz). This behavior suggests the absence of a stable, percolated filler network and is consistent with the viscous liquid-like response observed in the corresponding dynamic moduli results, where *G*″ exceeds *G*′. The emergence of a low-frequency Newtonian plateau indicates that flow behavior in this formulation is dominated by viscous dissipation rather than elastic energy storage, further confirming that excessive ZnO loading leads to network breakdown and enhanced polymer chain mobility.

The tensile properties of the PLA/PBS blend, the PLA/PBS/CF composite, and the ZnO-modified PLA/PBS/CF composites are summarized in Table 3. In addition to acting as stress concentrators, ZnO nanoparticle agglomerates adversely affect the interfacial adhesion between the PLA/PBS matrix and the carbon fibers, thereby diminishing the reinforcing efficiency of the hybrid filler system. Instead of contributing to effective load transfer, poorly dispersed ZnO clusters behave as structural defects, facilitating interfacial debonding and accelerating crack initiation and propagation under tensile loading. This microstructural deterioration explains the simultaneous reduction in Young’s modulus, tensile strength, and elongation at break observed at higher ZnO loadings, despite the intrinsically high stiffness of ZnO nanoparticles.

**Table 3:** Tensile Properties of PLA/PBS, PLA/PBS/CF, and ZnO-Added PLA/PBS/CF Composites

Sample	Tensile Stress at Maximum Force [MPa]	Tensile Strain at Maximum Force [%]	Tensile Strain at Break [%]	Tensile Stress at Break [MPa]	Modulus [MPa]
**PLA/PBS**	55.41	5.27	9.33	23.66	4252.09
**PLA/PBS/CF**	70.75	1.31	1.31	70.75	17203.81
**1 ZnO–CF**	55.17	1.66	1.66	55.17	13274.38
**3 ZnO–CF**	56.68	0.30	0.30	56.68	14252.83
**5 ZnO–CF**	42.70	0.18	0.18	42.70	10272.78
**7 ZnO–CF**	28.53	0.06	0.06	28.53	-

The negligible variation in tensile properties for the composite containing 1 wt % ZnO (Table 3) indicates that, at low nanoparticle content, ZnO remains sufficiently dispersed and does not significantly alter the stress distribution within the composite. Under these conditions, the reinforcing effect of carbon fibers dominates the mechanical response, and the ZnO nanoparticles neither enhance nor deteriorate the tensile performance to any measurable extent. In contrast, composites containing 3 wt % ZnO and above exhibit a pronounced degradation in mechanical performance, marked by a sharp decline in tensile strain at maximum force and at break. The drastic reduction in elongation at break at 5 and 7 wt % ZnO reflects severe embrittlement of the composite system. This embrittlement is attributed to restricted polymer chain mobility induced by rigid ZnO agglomerates and suppression of plastic deformation mechanisms within the PLA/PBS matrix. Furthermore, the clustered nanoparticles limit energy dissipation during deformation, resulting in premature brittle fracture behavior. The mechanical trends summarized in Table 3 are consistent with the rheological observations discussed above. At elevated ZnO loadings, the reduction in storage modulus and complex viscosity indicates the breakdown of an effective filler network in the melt state, which translates into inferior load-bearing capability in the solid state.^43,44,46^

### 3.5 Electromagnetic Interference Shielding Performance

Electromagnetic interference (EMI) shielding performance is primarily based on two mechanisms: reflection (SE_R_) and absorption (SE_A_). The sum of these two components constitutes the material’s total EMI shielding effectiveness (SE_T_). Figure 7a–d demonstrates the EMI shielding performance of PLA/PBS/CF-ZnO composites and the contributions of the SE_R_ and SE_A_ components to this performance. The PLA/PBS matrix exhibits SE_T_ values in the range of 0–3 dB across the entire frequency range. Due to its low electrical conductivity and limited dielectric polarization capability, the matrix is largely transparent to electromagnetic waves, which is consistent with the literature and expected for polymeric systems.^57^ In our research group’s previous studies and other findings reported in the literature, it has been demonstrated that incorporating carbon fiber into polymer matrices significantly improves overall EMI shielding performance.^30,41^ When 10%, 20%, and 30% CF were added to the PA11/PLA matrix, SE_T_ increased to 15, 23, and 28 dB, respectively.^30^ Xie et al. reported in their study that the pure PLA matrix showed almost no EMI shielding capability and exhibited only a very low EMI SE value of 0.5 dB. Adding CF to the PLA matrix significantly improved the shielding performance. EMI SE has increased to 13.7 dB for PLA/10CF, 27.6 dB for PLA/20CF, and 29.2 dB for PLA/30CF. However, they reported that increasing the CF content from 20% to 30% provided only limited improvement, with the EMI SE increase reaching saturation at this point and additional CF not proportionally improving shielding performance.^41^ Previous studies have reported that carbon fiber significantly improves EMI attenuation performance. Therefore, in this study, the CF ratio in the matrix composition was fixed at 20 wt %, which is widely reported in the literature as a near-optimal concentration for forming an effective conductive network without unnecessary filler excess, and different amounts of ZnO were added. Although the CF content was not varied in the present study, previous literature clearly demonstrates that increasing CF loading enhances EMI shielding performance due to the formation of a more efficient conductive network within the polymer matrix. As reported in earlier studies, increasing CF content from 10 wt % to 30 wt % leads to a significant improvement in EMI SE, primarily governed by reflection-dominant mechanisms associated with enhanced electrical conductivity.^30^ However, beyond a certain threshold (e.g., 20 wt %), the improvement tends to plateau due to the saturation of the conductive network. Therefore, the selection of 20 wt % CF in this study is considered an optimal balance point, allowing the additional contribution of ZnO to be more clearly evaluated, particularly in terms of absorption-dominated shielding behavior.

**Figure 7:**
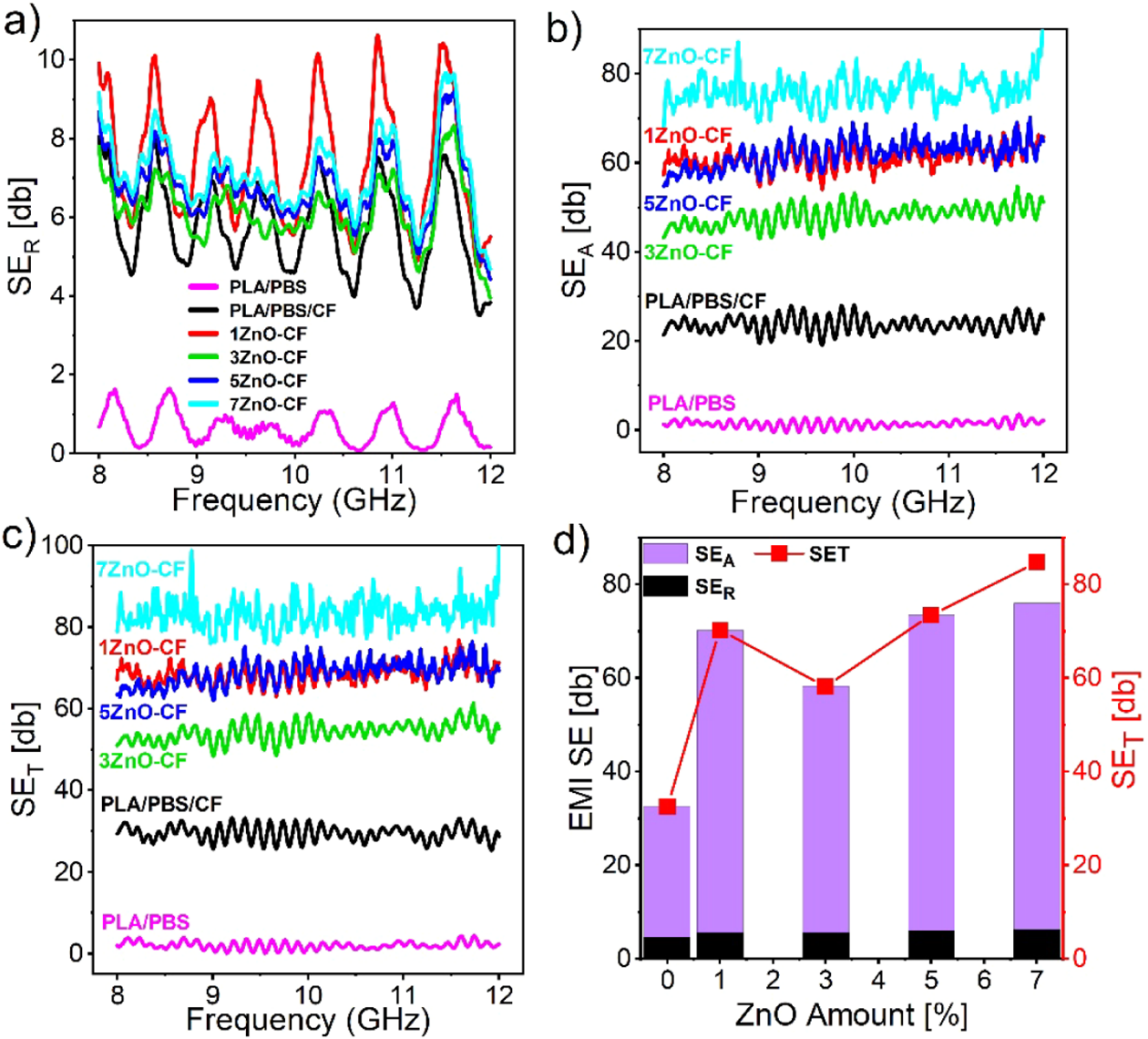
Variation of (a) SE_R_, (b) SE_A_, and (c) SE_T_ (EMI SE) of PLA/PBS/ZnO–CF composites with frequency in the X-band, and (d) comparison of SE_R_, SE_A_, and SE_T_ as a function of the ZnO amount at 10 GHz.

To enable a detailed evaluation, the individual contributions of reflection and absorption mechanisms to EMI SE are presented in Figure 7a and b. The low SE_R_ values observed in the 2–9 dB range in Figure 7a indicate that the composites exhibit good impedance matching with the void environment, confirming that reflection from the surface is limited. SE_R_ values showed a slight increase with the increase in ZnO content. However, the relatively low SE_R_ despite the ZnO content is due to the material surface not having a high reflectance capacity. Since carbon fiber and the polymer matrix do not exhibit metallic conductivity, a significant portion of electromagnetic waves penetrates the material and is absorbed rather than reflected off the surface. Figure 7b shows that, in fixed CF contents, the SEA value always remained above the SER value as the ZnO content increased. The fact that SEA is higher than SER indicates that the overall EMI shielding performance of PLA/PBS/CF-ZnO composites is primarily based on the absorption mechanism and that EM waves are largely attenuated within the material’s internal structure. In the study conducted by Waseem et al., increasing CF-ZnO content has been reported as the dominant mechanism at a rate above 80% SEA.^58^ This situation confirms that SE_R_ values remain relatively low and indicates that shielding behavior is essentially governed by the absorption mechanism. Furthermore, absorption-based EMI shielding is considered a preferred mechanism for preventing the formation of secondary electromagnetic interference caused by reflection. Therefore, due to the absence of metallic luster on the composite surface and the presence of numerous internal interfaces within the structure, SE_R_ remains limited; in contrast, SE_A_ emerges as the dominant and decisive mechanism in the attenuation of electromagnetic waves penetrating the matrix.

As shown in Figure 7c, the samples’ SE_T_ values are largely unaffected by frequency changes. The electromagnetic interference (EMI) shielding effectiveness (SET) of the PLA/PBS matrix alone is measured at 1.7 dB at 10 GHz. Adding 20 wt % carbon fiber (CF) to form the PLA/PBS/CF composite increases this to 30.7 dB at the same frequency, highlighting the role of CF’s conductive network in enhancing shielding. When ZnO nanoparticles are incorporated into the PLA/PBS/CF composite, the SET varies nonmonotonically with ZnO content: 70 dB for 1 wt %, 59 dB for 3 wt %, 73 dB for 5 wt %, and 85 dB for 7 wt %. This trend can be justified by the interplay between ZnO dispersion quality, concentration effects, and synergistic interactions with the CF network in EMI shielding mechanisms. At low ZnO loading (1 wt %), the nanoparticles achieve well dispersion within the PLA/PBS/CF matrix (see rheology results for more details). This optimal dispersion maximizes interfacial polarization, dielectric loss, and the formation of a hybrid conductive/dielectric percolating network, resulting in a high SET of 70 dB—more than double the 30.7 dB of the CF-only composite—with minimal filler addition. This dramatic enhancement at low ZnO loading is consistent with recent findings in CF-based hybrid systems, where small additions of ZnO nanoparticles have been shown to create dielectric bridges within the conductive fiber network, generating abundant heterogeneous interfaces that amplify interfacial polarization and multiple internal scattering far beyond additive filler contributions.^35,59–61^

Increasing the ZnO concentration to 3 wt % leads to agglomeration, which disrupts the uniform network, reduces the effective surface area for wave interactions, and potentially creates voids or weak points in the shielding structure, causing a dip in SE_T_ to 59 dB despite the higher loading. However, at even higher concentrations (5 and 7 wt %), the greater overall quantity of ZnO begins to compensate for the agglomeration effects by providing more abundant sites for electromagnetic wave absorption (Figure 7b), even if the dispersion is suboptimal. This leads to recovered and further improved SE_T_ values of 73 and 85 dB, respectively, as the sheer volume of ZnO overwhelms the dispersion limitations through denser filler packing.

Although it varies depending on application characteristics, EMI attenuation values of 20 dB and above are generally considered sufficient for practical applications.^59^ The results obtained are consistent with previous studies in the literature. Song et al. developed zinc oxide nanoparticle-modified mesoporous carbon spheres and demonstrated a 39.3 dB EMI SE at 10.4 GHz for ZnO/OMCS-30.^62^ Patle et al. prepared carbon foam derived from carbon fiber-reinforced phenolic resin decorated with nickel (Ni) and iron (Fe) nanoparticles and achieved the highest EMI SE of 62.3 dB with Fe nanoparticle-containing carbon fiber composite foam (Fe@C–CF).^63^ In the study conducted by Waseem et al., the EMI shielding effectiveness of CF-30% ZnO, CF-50% ZnO, and CF-70% ZnO reached 32.06, 38.08, and 34.69 dB, respectively.^58^

The amount of ZnO fills the pores and voids in the composite, enabling the formation of internal conductive pathways; thus, dielectric loss and multiple internal reflection mechanisms are activated. In regions enriched with ZnO nanoparticles, the interfaces surrounding the carbon fibers increase; EM waves travel a longer path by reflecting multiple times between these layers and are mostly absorbed. The highest SE_T_ reported for a composite containing 50% ZnO is ∼38 dB,^58^ this study, the PLA/PBS/CF composite containing 1% ZnO reached ∼70 dB, indicating that similar mechanisms are operating much more effectively. The difference can be explained by the optimized distribution of the carbon fiber-supported conductive network in the PLA/PBS matrix and ZnO, possibly due to the sample thickness and the specific production process. These values are well above the SE thresholds recommended in the literature for practical applications. ZnO doping triggers an increase in dielectric loss within the internal structure, which significantly improves overall shielding performance via SE_A_.

EMI protection typically uses practical thresholds of 20 and 30 dB. 20 dB SE corresponds to approximately 99% attenuation (damping); this value is acceptable for most commercial applications. Thirty dB indicates that over 99.9% of electromagnetic energy is blocked and is targeted in situations requiring higher protection.^59,64^ The average SE_T_ value of the 7% ZnO samples is ∼85 dB, which means that almost all incoming waves (≈99.999999%) are blocked. Therefore, the SE_T_ values obtained in the study (60–85 dB range) are well above the 20–30 dB thresholds and provide a high level of protection. In summary, thanks to the fixed CF and ZnO additions at different weight ratios, the composites exceed the shielding standards recommended in the literature; particularly with the dominance of SE_A_, the high decibel values provide reliable EMI protection in practical applications.

Figure 7d, a graph showing the individual contributions of SE_T_ components at a fixed frequency of 10 GHz, clearly demonstrates that the electromagnetic shielding performance of all composites is largely based on the absorption mechanism. The fact that SE_A_ constitutes 80% of the SE_T_ value in all composites indicates that absorption is clearly dominant within the electromagnetic-shielding mechanism. Zhou et al. reported in their study that SE_A_ dominates over SE_T_ at 10 GHz in PANI-doped carbon fiber-based composites.^65^ The dominance of SE_A_ over SE_T_ in all samples indicates that electromagnetic waves penetrating the material are largely attenuated through absorption; this also contributes to a certain degree of reduction in electromagnetic pollution caused by secondary reflections. The dominance of SE_A_ in composites is a result of both the polarization losses created by the ZnO additive and the conductive network and multiple scattering effects provided by carbon fibers. This behavior is also consistent with the criteria specified in the literature for high-performance absorption-dominant EMI-shielding materials. However, evaluating the EMI shielding mechanism of samples solely by comparing the SE_R_ and SE_A_ values is insufficient. Therefore, to evaluate the reflection, absorption, and transmission characteristics of incoming waves in EMI shielding behavior, relative parameters such as the reflection coefficient (*R*), absorption coefficient (*A*), and transmission coefficient (*T*) were calculated by deriving them from the measured S parameters.

Figure 8a shows the power coefficients (*A*, *R*, and *T*) as a function of the ZnO content at 10 GHz. The PLA/PBS matrix exhibits a high *T* value of 0.67, demonstrating a highly transparent structure to electromagnetic waves and exhibiting transparent behavior against EM waves.^66^ As ZnO is added and its amount increases, the *T* value decreases significantly, while the *R* and *A* values increase. The addition of ZnO to the composite increases the dielectric losses, facilitating the attenuation of EM waves. When the amount of ZnO exceeds a certain threshold, both *R* and *A* values stabilize, indicating that the electromagnetic response of the composite exhibits saturation behavior. Initially, the addition of ZnO increases dielectric losses and interfacial polarization, leading to an increase in *A*, while also causing an increase in *R* due to the mismatch in surface impedance. When the ZnO content exceeds the critical threshold, the additional dielectric contribution provided by the added particles to the system becomes limited, causing both *A* and *R* values to settle at a plateau level independent of the filler amount, exhibiting a stable behavior.

**Figure 8:**
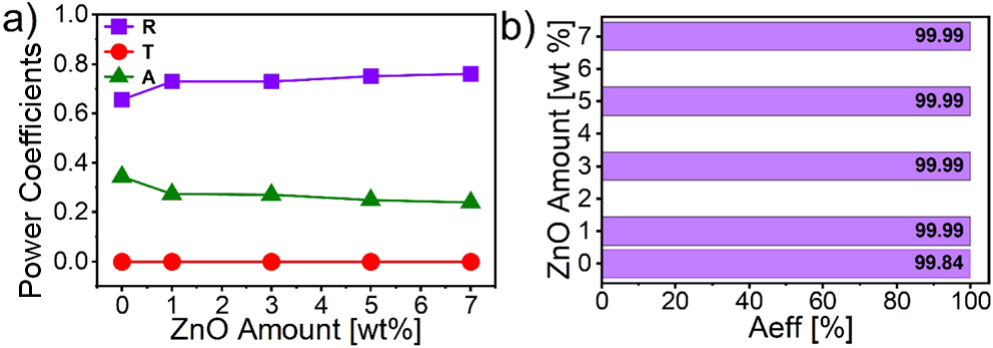
Power coefficients as a function of (a) the ZnO amount and (b) Effective absorbance.

Since the screening behavior in all samples was absorption-dominant, the absorption efficiency *A*_eff_ was calculated, and the results obtained are presented in Figure 8b. To evaluate the absorption potentials of the composites more accurately, the absorption efficiency was analyzed as a function of the ZnO content, and the variation in *A*_eff_ values was examined in this context. The *A*_eff_ value of the pure matrix (PLA/PBS) was 29%, the addition of CF increased this value to 99.84%, and the addition of ZnO enhanced to 99.99%. This demonstrates that CF and ZnO significantly enhance the matrix’s capacity to absorb EM waves. PLA/PBS/ZnO–CF composites have been observed to increase the absorption capacity of EM waves up to 99.99%, a performance that exceeds the standard requirements set for commercial applications.

To benchmark the EMI shielding performance of PLA/PBS/CF/ZnO composites developed in this study, a comprehensive comparison with recently reported polymer-based EMI shielding materials is presented in Table 4. As can be observed, the SE_T_ values achieved in this work (70–85 dB) significantly surpass those of most biodegradable and nonbiodegradable polymer composites reported in the literature. For instance, PLA/PEG/MWCNT nanocomposites achieved ∼42 dB, PLA/TPU/carbon black composites reached ∼27 dB, and ZnO-decorated carbonated cotton fiber composites exhibited 32–38 dB. The superior performance of our composites is attributed to the synergistic interplay between the conductive CF network and the dielectric loss enhancement provided by uniformly dispersed ZnO nanoparticles within the biodegradable PLA/PBS matrix. Moreover, the absorption-dominant shielding mechanism (>80% SE_A_) distinguishes these composites from many reflection-based systems, making them particularly suitable for practical EMI mitigation where secondary reflection must be minimized.

**Table 4:** Comparison of EMI Shielding Performance of PLA/PBS/CF/ZnO Composites (This Work) with Recently Reported Polymer-Based EMI Shielding Materials in X-Band Frequency

Composite System	Matrix Type	Filler(s)	Filler Loading	EMI SE (dB)	Dominant Mechanism	Ref.
**PLA/PBS/CF/ZnO**	**PLA/PBS** (80/20)	**CF + ZnO NPs**	**20 wt % CF + 1–7 wt % ZnO**	**70–85**	**Absorption (>80%)**	**This work**
PA11/PLA/CF	PA11/PLA	Carbon fiber	10–30 wt % CF	15–28	Reflection	^30^
PLA/SCF + CNT	PLA	Short CF + CNT	20 wt % CF + 1 wt % CNT	∼52	Absorption	^39^
PLA/PEG/MWCNT	PLA/PEG	MWCNT	4 wt %	∼42	Absorption	^67^
PLA/TPU/CB	PLA/TPU	Carbon black	30 wt %	∼27	Absorption	^55^
PCL/p-MWCNT	PCL	Pristine MWCNT	3 wt %	∼32	Absorption	^68^
PLA/GNP	PLA	Graphene nanoplatelets	15 wt %	∼15	Reflection	^69^
PLLA/PDLA/CNT	PLA	CNT	1 wt %	∼35.5	Absorption	^70^
ZnO/OMCS-30	Paraffin wax	ZnO/porous C spheres	–	∼39.3	Absorption	^57^
CF-ZnO/cotton	Carbonated cotton	CF + ZnO	30–70 wt % ZnO	32–38	Absorption (>80%)	^59^
PLA/CNT/MXene	PLA	CNT + Ti3C2Tx MXene	Various	∼39	Absorption	^71^
PLA/CF foam	PLA (foamed)	Carbon fiber	20 wt % CF	∼27–30	Absorption	^38^
Fe@C–CF foam	Phenolic resin	CF + Fe NPs	–	∼62.3	Absorption	^58^

## 4 Conclusion

The present study demonstrates that outstanding EMI shielding effectiveness can be achieved in biodegradable PLA/PBS-based composites through the strategic hybridization of carbon fibers and ZnO nanoparticles, with the shielding performance strongly governed by ZnO loading and dispersion quality. The incorporation of 20 wt % carbon fiber established a stable conductive network, increasing the SE_T_ from 1.7 dB for the neat PLA/PBS matrix to 30.7 dB. The subsequent addition of ZnO nanoparticles led to a substantial enhancement in EMI shielding, primarily through absorption-driven mechanisms.

Among all formulations, the composite containing 1 wt % ZnO emerged as the optimal system, delivering a high SE_T_ of approximately 70 dB while preserving the structural integrity and rheological stability. At this low loading, ZnO nanoparticles were effectively dispersed within the PLA/PBS/CF matrix, as indirectly confirmed by rheological measurements showing a significantly enhanced storage modulus and complex viscosity. This uniform dispersion maximized interfacial polarization, dielectric loss, and multiple internal scattering of electromagnetic waves, enabling highly efficient shielding with a minimal nanoparticle content.

Although higher ZnO loadings (5 and 7 wt %) resulted in even greater absolute SE_T_ values, reaching up to ∼85 dB, these improvements were accompanied by clear drawbacks, including nanoparticle agglomeration, deterioration of the filler-supported network, reduced melt elasticity, lower mechanical performance, and increased embrittlement. The nonmonotonic trend in SE_T_—with a decrease at 3 wt % ZnO—highlights the critical role of dispersion quality over filler quantity in governing EMI shielding efficiency. Excessive ZnO loading disrupts effective interfacial interactions and compromises the multifunctional performance of the composite, even when shielding values remain high.

Importantly, all CF–ZnO hybrid composites exhibited absorption-dominant EMI shielding behavior, with SE_A_ accounting for more than 80% of the total shielding effectiveness. This absorption-driven mechanism is particularly advantageous for practical EMI protection, as it minimizes secondary electromagnetic reflection and associated interference. Overall, the results demonstrate that incorporating a low ZnO content (1 wt %) yields the most effective and well-balanced EMI shielding performance, presenting a lightweight and sustainable solution for biodegradable polymer-based shielding systems. The findings further confirm that fully biodegradable composites can match or even outperform traditional nondegradable EMI shielding materials, highlighting a promising and environmentally responsible approach for electromagnetic protection in electronics, aerospace, and emerging 5*G*/6G technologies.
